# Altered dendritic morphology of MEC II pyramidal and stellate cells in Rett syndrome mice

**DOI:** 10.3389/fnana.2025.1580435

**Published:** 2025-06-24

**Authors:** Manigandan Krishnan, Ayishal B. Mydeen, Mohammed M. Nakhal, Marwa F. Ibrahim, Richard L. Jayaraj, Milos R. Ljubisavljevic, Mohammad I. K. Hamad, Fatima Y. Ismail

**Affiliations:** ^1^Department of Pediatrics, College of Medicine and Health Sciences, United Arab Emirates University, Al Ain, United Arab Emirates; ^2^Department of Anatomy, College of Medicine and Health Sciences, United Arab Emirates University, Al Ain, United Arab Emirates; ^3^Department of Physiology, College of Medicine and Health Sciences, United Arab Emirates University, Al Ain, United Arab Emirates; ^4^Department of Neurology (Adjunct), Johns Hopkins University School of Medicine, Baltimore, MD, United States

**Keywords:** Rett syndrome, Mecp2 mouse model, dendritic branching, pyramidal cells, stellate cells, medial entorhinal cortex

## Abstract

**Introduction:**

Mutations in the methyl-CpG-binding protein-2 gene (*MECP2*), which cause Rett syndrome (RTT), disrupt neuronal activity; however, the impact of the *MECP2* loss-of-function on the cytoarchitecture of medial entorhinal cortex layer II (MECII) neurons—crucial for spatial memory and learning—remains poorly understood.

**Methods:**

In this study, we utilized Golgi staining and neuron tracing in the *Mecp*2^+/−^ mouse model of RTT to investigate the pyramidal and stellate cell alterations in MECII.

**Results and discussion:**

Our findings revealed that pyramidal cells displayed a significant reduction in apical dendritic length, soma size, and spine density, while basal dendrites showed increased dendritic complexity and branching. On the other hand, stellate cells exhibited dendritic hypertrophy along with increased soma size, primary dendrites, and localized increase in dendritic intersections, despite an overall reduction in total dendritic length and spine density. These findings underscore the notion that *MECP2* loss-of-function can disrupt MECII pyramidal and stellate cell cytoarchitecture in a cell-type-specific manner, emphasizing its critical role in maintaining proper dendritic morphology in circuits, which is crucial for learning and memory.

## 1 Introduction

Dendrites are specialized neural structures that receive and integrate synaptic inputs from presynaptic neurons, playing a crucial role in shaping the neuronal networks that underpin cognitive and behavioral processes (Wu et al., 1999; Prigge and Kay, 2018; Hamad et al., 2023). Dysregulation in dendritic architectures such as length, branching patterns, and spine density can impair synaptic networks and lead to region- and cell-type-specific neuronal dysfunction, which in turn influences cognitive and behavioral traits (Armstrong et al., 1995; Moretti et al., 2005; Valnegri et al., 2015). Many neurodevelopmental diseases, such as autism spectrum disorder (ASD) (Geschwind and Levitt, 2007), fragile X syndrome (Contractor et al., 2015), trisomy 21 Down syndrome (Klein and Haydar, 2022), and epilepsy (Leifeld et al., 2022), are associated with unusual changes in the structure of dendrites. Among these ailments is RTT, a severe and progressive condition caused by X chromosome *MECP2* gene mutations (Amir et al., 1999; Gulmez Karaca et al., 2019; Good et al., 2021), with an estimated frequency of 1 in 10,000. RTT almost entirely affects women (Hagberg, 1985; Neul et al., 2010) and is characterized by cognitive, learning, memory, and movement disabilities (Armstrong, 2005; Guy et al., 2007).

Maintaining dendritic structure and function across growth and development depends on the *Mecp2* protein, a transcriptional regulator in neurons (Shahbazian et al., 2002; Gonzales and LaSalle, 2010; Neul et al., 2010). RTT syndrome patients have varied and region-specific dendritic structural alterations, especially in layers 2, 3, and 5 of the motor cortex, which and prefrontal cortex exhibit the most marked decrease in dendritic complexity and spine density (Leontovich et al., 1999), however, less dramatic alterations are seen in the hippocampal and visual cortex (Belichenko et al., 1997; Armstrong, 2005). Mouse models of *MECP2* mutations recapitulate these region-specific alterations. *Mecp2*-null mice showed a significant decrease in the branching of dendrites and the number of spines on pyramidal neurons in the somatosensory (Layers 2, 3, and 5) and motor (Layer 5) areas of the brain (Fukuda et al., 2005). In the *Mecp*2^T158A/y^ partial mutation models, older animals showed changes in the dendrites of the somatosensory area and smaller cell bodies in the sensorimotor cortex, which were connected to later behavioral issues (Wang et al., 2013).

The brain region crucial for memory is the entorhinal cortex (EC), which acts as an interface between the neocortex and the hippocampus. The MECII, which is a part of the EC, has six layers (L1 to L6), and these layers play a major role in processing spatial and sensory inputs for navigation and memory formation (Howard et al., 2014). The MECII has two different cell types—pyramidal and stellate cells—which differ not only in their gene and protein expression markers but also in their structural characteristics and projection targets (Alonso and Klink, 1993). MECII pyramidal cells selectively express calbindin (a protein that binds calcium) and Wolfram syndrome 1 (Wfs1)—a gene involved in endoplasmic reticulum homeostasis. They send signals mainly to the hippocampal CA1 area and mostly to other parts of the cortex and subcortex (Brun et al., 2008; Kitamura et al., 2014, 2017). Whereas MECII stellate cells typically express the transcription factor reelin, primarily innervating dentate gyrus-granular cells and hippocampal CA3 pyramidal cells, thereby playing a vital role in spatial memory and contextual learning (Kitamura et al., 2014; Ray et al., 2014; Hamad et al., 2021). Research reported that MECII are among the earliest neurons affected by Alzheimer's disease (AD) by disrupting the link between the neocortex and hippocampus, contributing to early memory loss (Khan et al., 2014).

Given RTT-associated cognitive deficits and the MECII's role in spatial and memory circuits, we hypothesize that *MECP2* loss-of-function might lead to cell-type-specific dendritic alterations in MECII, potentially contributing to the pathophysiology of RTT phenotypes. Therefore, we aimed to investigate how loss of *MECP2* affects the changes in the dendrites of MECII pyramidal and stellate cells by using a *Mecp*2^+/−^ mouse model. Dendritic architecture was assessed using Golgi staining, high-resolution imaging, and morphometric analysis to better understand how *MECP2* loss leads to the cell-type-specific disruptions in MECII.

## 2 Material and methods

### 2.1 Animals and maintenance

Heterozygous female *Mecp2*^+/−^ mice (8 weeks of age, strain: 003890, B6.129P2(C)- *Mecp2*^*tm*1.1*Bird*^*/J*) were obtained from Jackson Laboratories (Bar Harbor, ME, USA). The 8-week-old wild-type (WT) C57BL/6 of both sexes were procured from the Laboratory Animal Research Facility at the United Arab Emirates University. All experimental mice were housed in a climate-controlled environment with a 12-h light/dark cycle and had unrestricted access to food and water. The temperature was maintained at 25°C.

### 2.2 Experimental design

We selected 12-month-old female *Mecp2*^+/−^ mice (*n* = 6) that consistently exhibited hind-limb clasping (Supplementary Figures 1D, E) for subsequent morphological analysis of the MECII region. Respective age-matched WT female mice (*n* = 6) were used as normal controls. The animal experimental protocols were approved by the Animal Use Ethics Committee of the United Arab Emirates University (ethical approval number: ERA_2021_8444).

### 2.3 Neuronal dendrite golgi-cox staining

The morphology of neuronal dendrites was analyzed using the FD Rapid GolgiStain Kit (FD NeuroTechnologies, Inc.; PK401 Cell Systems Biology). Tissue preparation and staining were performed according to the manufacturer's instructions. At the end of the experiment, the body weights of WT and *Mecp2*^+/−^ controls were recorded. Mice were decapitated, their brains were gently washed with water, weighed, and then incubated with equal volumes of Golgi-Cox impregnation solution (Solutions A and B) for 2 weeks in the dark. Subsequently, the brains were immersed in Solution C for 1 week. A vibratome was utilized to obtain 120 μm parasagittal sections that were cut from both hemispheres, extending to the medial border of the MEC region. The absence of a broad white band surrounding the external capsule was a distinguishing characteristic of these sections. A maximum of 16 slices were obtained per mouse, including the MEC region from both hemispheres. The brain slices were then rehydrated in milli Q water, stained with Solutions D and E for 10 min, washed again in milli Q water, dehydrated, and cover slipped with a water-based mounting medium.

**Figure 1:**
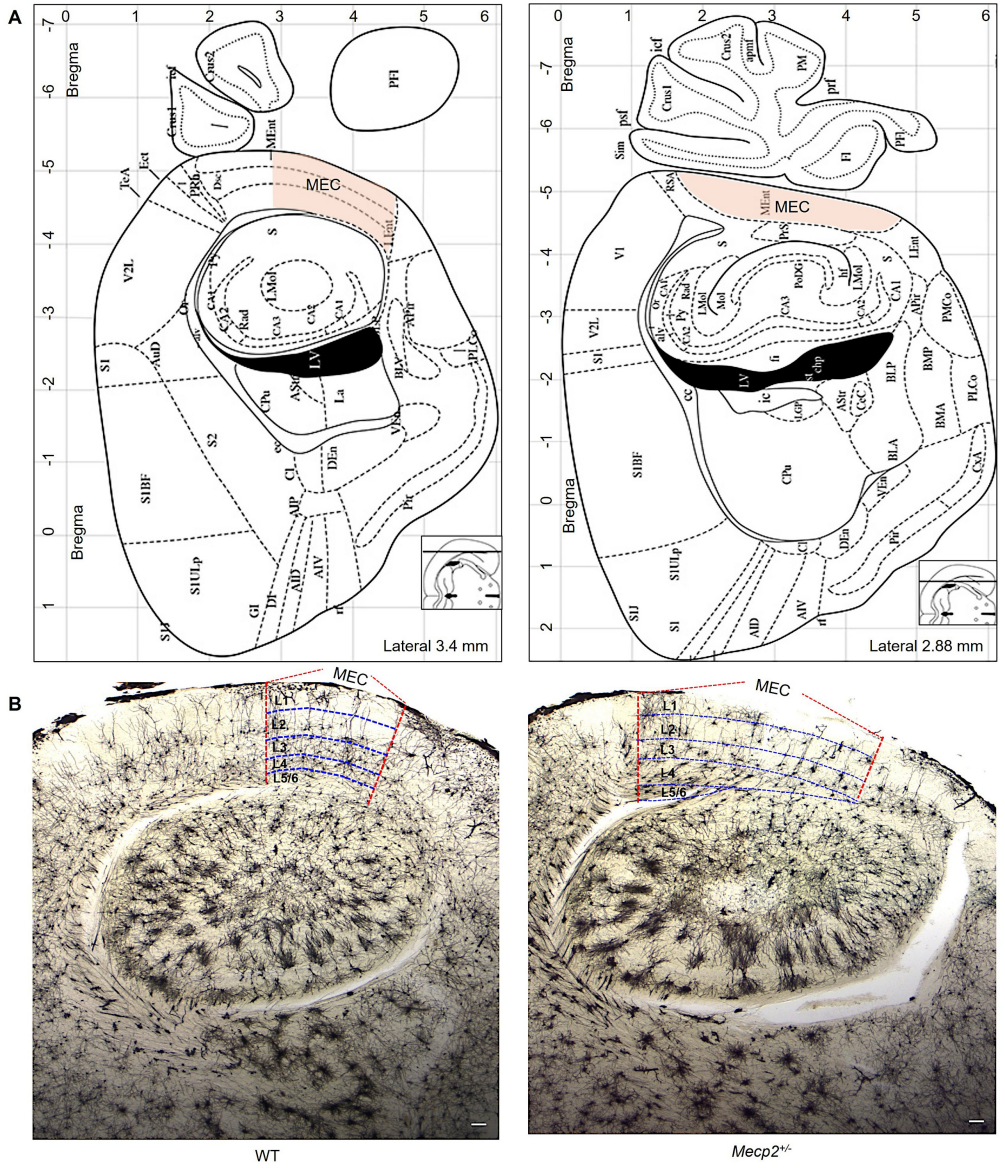
The MEC area is utilized to measure dendritic morphology and trace neurons. **(A)** The MEC (orange hue) area utilized to select neurons for dendritic morphology evaluations was established using the mouse brain atlas (Paxions, 2008). **(B)** Golgi staining shows dendritic arbor structures and branching patterns in sagittal slices of WT and *Mecp*2^+/−^ mice (2.5× magnification, scale bar: 1000 μm). Vertical lines (red color) represent MEC areas, and horizontal lines (blue color) indicate MEC layers (L1 to L6), as established by previous findings (Witter et al., 2017).

The MEC area for morphological assessment was defined using the mouse brain atlas (Paxions, 2008). For WT mice, off-sagittal sections were aligned to the following Bregma coordinates: anteroposterior (AP) = −5.0 mm, mediolateral (ML) = +3.5 mm, and dorsoventral (DV) = +3.0 mm (Figures 1A, B) and for *Mecp2* mice, the sections were aimed at the Bregma coordinates: AP = −5.0 mm, ML = +2.9 mm, and DV = +3.0 mm (Figures 1A, B). These locations were chosen to uniformly target the MEC in all animals. To identify the layers (L1 to L6) within the MEC, we followed cytoarchitectural criteria as described previously (Canto et al., 2008; Witter et al., 2017), which separate layers according to cell density, soma size, and general cytoarchitecture. L1 consists of multipolar neurons (MPNs). Dense clusters of tiny stellate neurons are defined in L2; a narrow band of pyramidal neurons are defined in L2 and L3; moderately spiny pyramidal-shaped neurons in L4; more widely dispersed bigger pyramidal cells are defined in L5 and MPNs are located throughout L6.

### 2.4 Identification and 3D reconstruction of pyramidal and stellate cells

In this study, pyramidal and stellate cells from MECII regions were identified and reconstructed using the Neurolucida™ system (MicroBrightField, MBF Bioscience Inc., Williston, VA, USA) at 1000 × magnification, following previously described methods (Hamad et al., 2024). A detailed analysis of the morphology of each neuron was conducted to characterize the architecture of the dendrites and the projections of the axons. Pyramidal cells were identified by their unique apical dendrite and several small basal dendrites. These dendrites are highly spiny, resembling those of pyramidal cells in the somatosensory and motor cortices. To quantify their morphological parameters, soma size, apical dendritic length, mean basal dendritic length (total basal dendritic length divided by the number of primary dendrites), total apical dendritic segments, mean basal dendritic segments, and dendritic spine density (number of spines/10 μm of dendrites) were analyzed.

Stellate cells were recognized by a larger number of primary dendrites, high dendritic spine density, and axonal projections extending to the white matter rather than forming local branches. Although these neurons exhibit a multipolar arrangement, they differ markedly from inhibitory interneurons, which typically branch locally (Karube et al., 2004) and possess four primary dendrites with relatively few spines (Kawaguchi et al., 2006). In contrast, stellate cells project their axons distally within MECII and toward the white matter and hippocampus (Kitamura et al., 2014; Fuchs et al., 2016), while possessing eight spiny primary dendrites. To quantify their morphological parameters, we calculated soma size, dendritic spine density (number of spines/10 μm of dendrites), mean dendritic length (total dendritic length divided by the number of primary dendrites), mean number of dendritic segments (total dendritic segments divided by the number of primary dendrites), and the number of primary dendrites.

Sholl analysis was used to assess dendritic complexity in both stellate and pyramidal cells in MECII of *Mecp*2^+/−^ mice, as compared to wild-type (WT) controls. This approach involves drawing concentric circles (or spheres) at 10 μm intervals around the soma and counting the number of dendritic intersections. This methodology facilitates the identification of specific regions where dendritic complexity changes (Sholl, 1953; Zagrebelsky et al., 2020).

### 2.5 Statistical analysis

All statistical analyses were conducted using SigmaPlot Version 12. Comparisons between the two groups were performed using Student's unpaired *t*-test when the normality test (Shapiro-Wilk) was passed; otherwise, the Mann–Whitney test was used. Data are presented as the mean ± standard error of the mean (SEM). Results were considered statistically significant at *p* < 0.05.

## 3 Results

### 3.1 *Mecp2^+/−^* mice show altered dendritic morphology of MECII pyramidal cells

**Figure 2:**
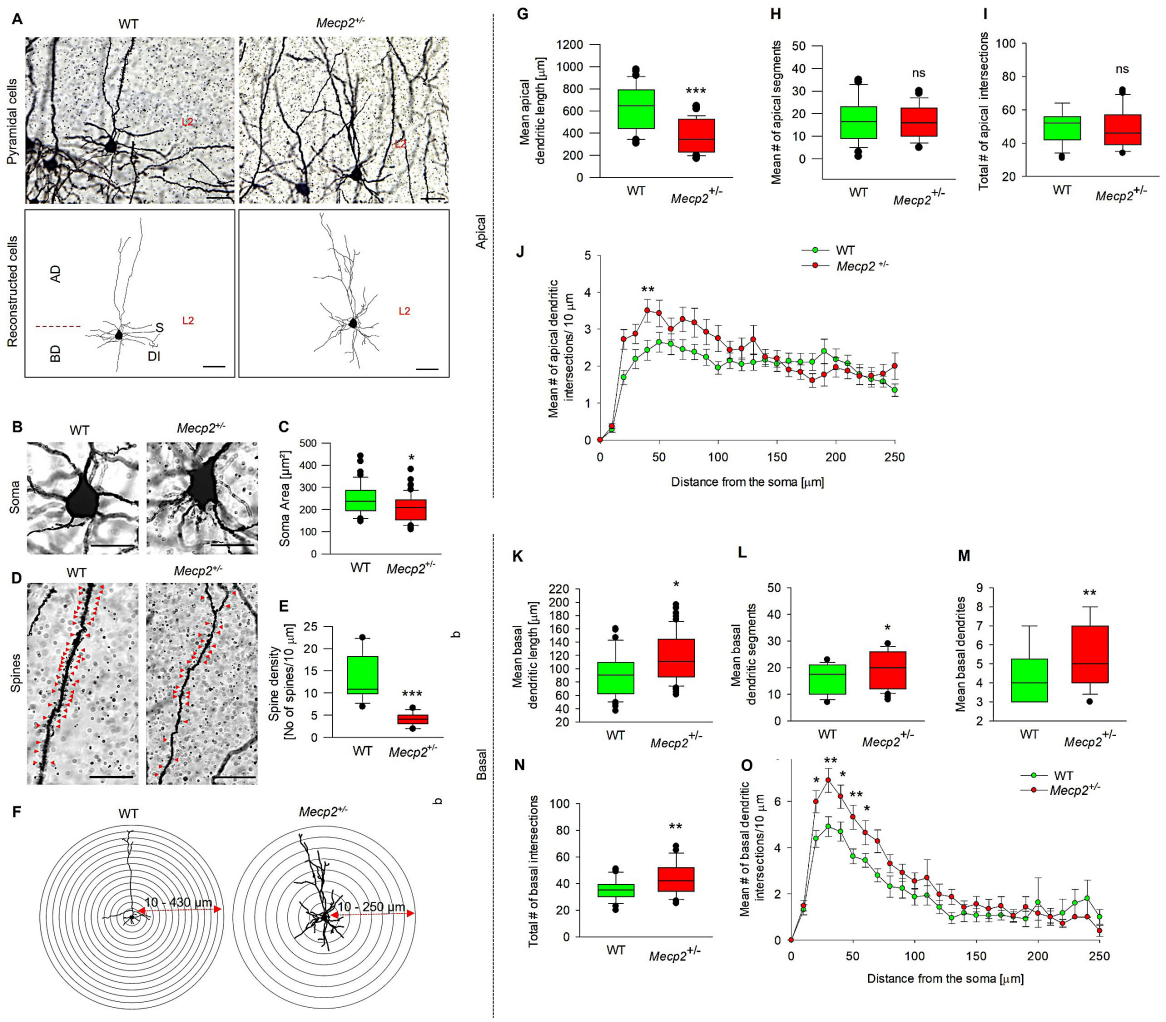
Quantitative morphometric analysis of dendrites in MECII pyramidal cells of *Mecp*2^+/−^ mice. **(A)** Golgi staining reveals dendritic arbor structures and branching patterns of pyramidal cells in sagittal sections of WT and *Mecp*2^+/−^ mice (40× magnification, scale bars: 100 μm), together with their corresponding 2D reconstructed pyramidal neuron images. AD-apical dendrites, BD-basal dendrites, S-soma, and DI-dendritic intersections, and L2-Layer2. Golgi-stained image showing **(B)** Soma size and **(C)** mean soma area, **(D)** spine distributions and **(E)** dendritic spine densities of WT and *Mecp*2^+/−^ mice. Representative stereotype images were obtained with a 63× oil immersion objective (20 μm, mean ± SEM (*n* = 6), number of cells analyzed = 12 cells/group, *p < 0.05, ***p < 0.001 by Mann-Whitney *t*-test). **(G)** Mean apical dendritic length, **(H)** mean apical dendritic segments, and **(I, J, F)** Sholl analysis of dendritic intersections in *Mecp*2^+/−^ mice compared to WT. Concentric circles are centered on the soma and spaced at 30 μm intervals, ranging from 10 to 430 μm for WT and 10 to 250 μm for *Mecp*2^+/−^, and **(K)** Quantitative analysis of mean basal dendritic length, **(K)** Mean basal dendritic segments, **(L)** Mean basal dendrites, **(M)** and **(N, O, F)** Basal dendritic intersections in *Mecp*2^+/−^ mice compared to WT. Data are presented as mean ± SEM (*n* = 6 independent preparations; number of cells analyzed/group = 42 cells (WT) and 53 cells (*Mecp*2^+/−^), *p < 0.05, **p < 0.01, ***p < 0.001, ns non-significant by Mann-Whitney *t*-test).

We chose to study the morphology of MECII neurons in the *Mecp*2^+/−^ mouse model because MECII plays a crucial role in connecting the hippocampus with other cortical regions that are essential for spatial navigation and memory (Howard et al., 2014). As shown in Figures 2A, G, *Mecp*2^+/−^ mice exhibited a significant reduction in mean apical dendritic length (381.41 ± 24.26 μm) when compared to WT (563.1 ± 38.66 μm), but the number of mean apical dendritic segments (Figure 2H) remained unchanged in *Mecp*2^+/−^ (17.35 ± 1.18), and WT mice (16.9 ± 1.41). To determine in which dendritic compartment of the apical dendrites the reduction of complexity has occurred, we performed Sholl analyses, which revealed an increase in mean apical dendritic intersections specifically at 40 μm (3.18 ± 0.26) from the soma in *Mecp*2^+/−^ mice (Figures 2F, I, J) when compared to WT (2.42 ± 0.27 at 40 μm). The total number of apical intersections remained unchanged.

Considering the apical dendritic changes observed in pyramidal cells of *Mecp*2^+/−^ mice, we further investigated the dendritic patterns in the basal region of these neurons. Figure 2A depicts our qualitative morphometric study of basal dendrites in pyramidal cells, which indicated substantial alterations in the dendritic structure of *Mecp*2^+/−^ mice. *Mecp2* heterozygosity resulted in a longer mean basal dendritic length (115.72 ± 5.56 μm, Figure 2K) compared to WT (97.73 ± 6.34 μm). In addition, we noticed a significant increase in the mean basal dendritic segments (20.62 ± 1.36, Figure 2L), mean basal dendrites (5.50 ± 0.23, Figure 2M), and total number of basal dendritic intersections (49.25 ± 3.68, Figure 2N) compared to WT (mean basal dendritic segments, 5.76 ± 0.96; mean basal dendrites, 4.61 ± 0.29; and total number of basal dendritic intersections, 33.73 ± 2.11). Sholl analysis of dendritic complexity revealed that *Mecp*2^+/−^ mice exhibited increased basal dendritic intersections starting at 20 μm from the soma and extending to 60 μm (Figures 2F, O). Given the deficiencies in pyramidal cell development and differentiation in *Mecp*2^+/−^ mice, we next examined soma size and spine density patterns. Figures 2B, C indicate that *Mecp*2^+/−^ animals have a considerably reduced soma area (208.9 ± 8.47 μm^2^) compared to WT mice (245.3 ± 10.59 μm^2^). Spine distribution and density quantification results (Figures 2D, E) revealed a substantial drop in spine numbers (4.11 ± 0.39 spine density/10 μm) in *Mecp2*^+/−^ animals, compared to WT mice with 13.16 ± 1.50 spine density/10 μm. These morphological alterations in MECII pyramidal cells in *Mecp2*^+/−^ mice might contribute to altered synaptic connectivity.

### 3.2 Mecp2+/– mice show increased dendritic morphology of MECII stellate cells

**Figure 3:**
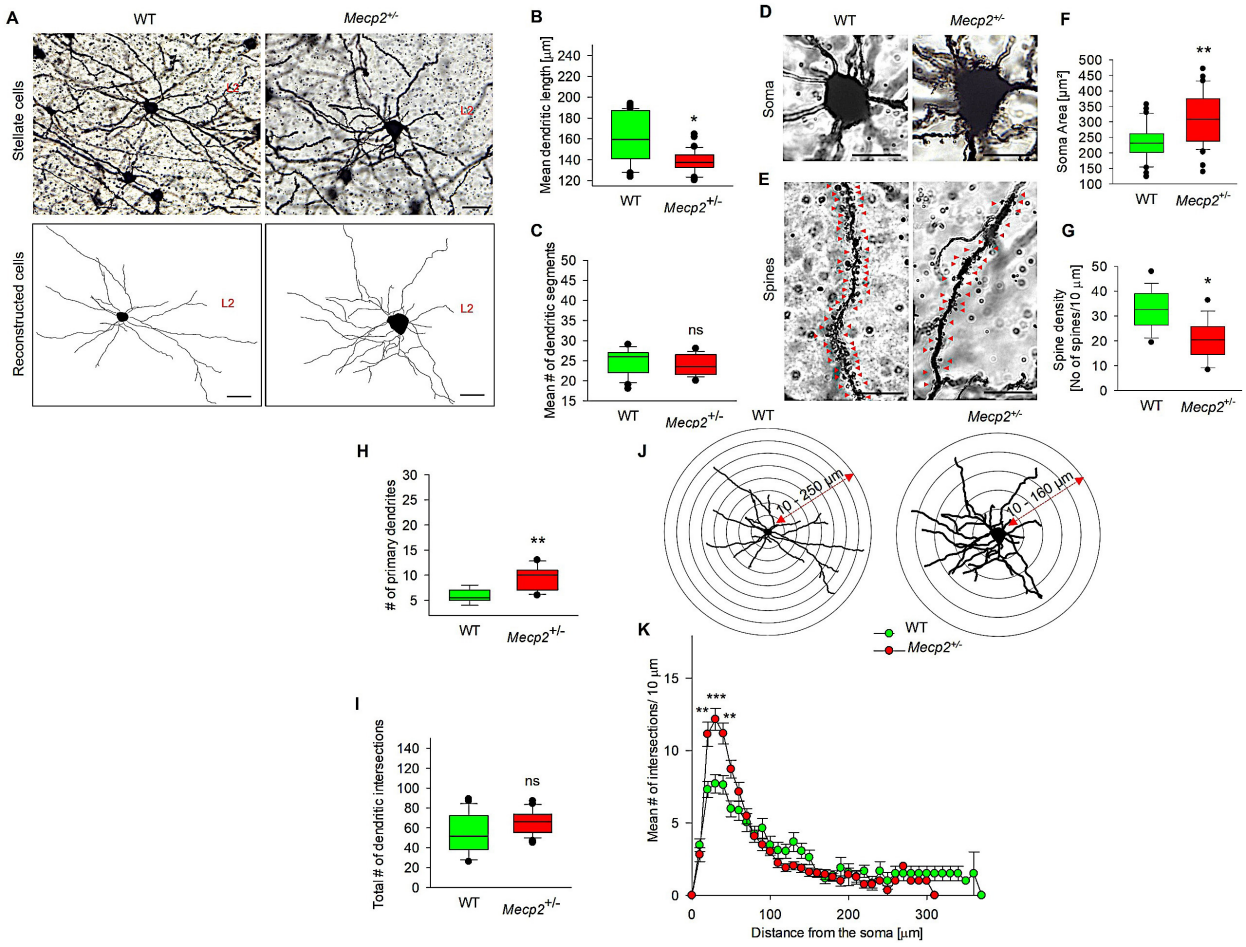
Quantitative morphometric analysis of dendrites in MECII stellate cells of *Mecp*2^+/−^ mice. **(A)** Representative Golgi staining of stellate cells in both WT and *Mecp*2^+/−^ mice, along with their corresponding 2D reconstructed images, showed variations in dendritic phenotypes (40× magnification, scale bar: 100 μm, L2: Layer 2). **(B)** Mean dendritic length, **(C)** Mean dendritic segments. Golgi-stained image showing **(D)** Soma size and **(E)** Spine distributions, **(F)** Mean soma area, and **(G)** dendritic spine densities of WT and *Mecp*2^+/−^ mice. Representative stereotype images of soma and spine densities were obtained with a 63× oil immersion objective (20 μm, mean ± SEM (*n* = 6), number of cells analyzed = 12 cells/group, *p < 0.05, **p < 0.01 by Mann-Whitney *t*-test). **(H)** Mean primary dendrites, **(I)** Total dendritic intersections, **(J)** Sholl analysis of dendritic intersections in the stellate cells of WT and *Mecp*2^+/−^ mice. Concentric circles are centered on the soma and spaced at 30 μm intervals, ranging from 10 to 250 μm for WT and 10 to 160 μm for *Mecp*2^+/−^ and **(K)** Sholl analysis showing increased dendritic intersections in *Mecp*2^+/−^ mice at 20–50 μm from the soma. Data are presented as mean ± SEM (*n* = 6 independent preparations); number of cells analyzed/group = 38 cells (WT) and 40 cells (*Mecp*2^+/−^); Comparisons: *p < 0.05, **p < 0.01, ***p < 0.001, and ns non-significant by the Mann-Whitney *t*-test with *Mecp*2^+/−^ mice).

We then explored the effect of *Mecp*2^+/−^ on the dendritic patterns of stellate cells (Figure 3), which are part of the grid cell network within the MECII and are also implicated in spatial navigation and memory. Figures 3A, B show that *Mecp2* heterozygosity was associated with a reduction in the mean dendritic length of stellate cells to 130.55 ± 5.95 μm from that of WT (138.61 ± 7.67 μm), while mean dendritic segments (Figure 3C) remained unchanged in both groups (*Mecp*2^+/−^, 25.24 ± 0.89; WT, 26.56 ± 1.63). However, dendritic hypertrophy was observed in *Mecp*2^+/−^ (Figure 3H), as indicated by an increased number of primary dendrites (9.84 ± 0.92) when compared to WT (5.21 ± 0.38). Furthermore, Sholl analysis of dendritic complexity revealed a distance-specific increase in mean dendritic intersections in *Mecp*2^+/−^ mice at 20 μm from the soma, extending to 50 μm (Figures 3J, K). However, the total number of dendritic intersections in MECII stellate cells (Figure 3I) did not differ between *Mecp*2^+/−^ (77.61 ± 4.24) and WT mice (65.62 ± 5.77). Notably, we observed severe dendritic alterations in MECII stellate cells. Further, we investigated the soma size and spine density changes in the *Mecp*2^+/−^ mice and observed that soma size significantly increased to 311.3 ± 13.31 μm (Figures 3D, F) compared to WT (236.8 ± 9.95 μm). Spine distribution and density analyses (Figures 3E, G) showed a decrease in the spine numbers in *Mecp*2^+/−^ mice (20.54 ± 2.38 spine density/10 μm) compared to the WT (32.75 ± 2.46 spine density/10 μm). Collectively, *Mecp2* loss-of-function can result in structural abnormalities in MEC stellate cells on L2, as evidenced by dropped spine density and altered dendritic structures.

## 4 Discussion

Loss-of-function in the *MECP2* gene produces RTT, a neurodevelopmental condition characterized by extensive anatomical and functional deficits in the brain. Aberrant neuronal structures are one of the suggested underlying mechanisms of RTT pathology (Chapleau et al., 2009; Albizzati et al., 2024). However, these abnormalities may vary substantially and are often cell-type and region-specific (Asaka et al., 2006; Smrt et al., 2007; Katz et al., 2012; Wang et al., 2013).

*Mecp2* mutant animal models and brain slices from RTT patients (Bauman et al., 1995; Guy et al., 2001, 2007) have consistently shown dendritic abnormalities such as decreased size, dendritic complexity, and spine density in the motor, frontal, temporal, visual, hippocampus, and somatosensory areas (Dunn and MacLeod, 2001; Moretti et al., 2005; Belichenko et al., 2009; Chapleau et al., 2009; Zhang et al., 2016). Though the dendritic pathology in these brain regions is well-documented, much less is known about MECII, a region vital for memory and sensory integration, and it is anatomically linked to both the hippocampus and other cortical regions (Kitamura et al., 2015, 2017; Witter et al., 2017; Osanai et al., 2023). Thus, an investigation into how *MECP2* affects the normal architectures of MECII pyramidal and stellate cells could provide deeper information about the structural deficits linked to RTT pathophysiology could pave the way for targeted therapeutic strategies. RTT animal models with inducible *MECP2* gene loss exhibit global brain shrinkage and a reduction in neuronal cell body size at late juvenile or adult stages (Nguyen et al., 2012). We observed a significant decrease in body and brain weight in *Mecp*2^+/−^ mice at 12 months of age, which aligns with postmortem reports of reduced brain weight in RTT patients. These reductions are thought to result from halted halted development or neuronal shrinkage, rather than neuronal degeneration (Armstrong et al., 1999).

In this study, we systematically examined the morphology of selected neuronal populations (such as pyramidal and stellate cells) in the MECII region of *Mecp*2^+/−^ mice exhibiting significant hind limb clasping (a RTT pathology), which is consistent with the reported motor phenotype in *MECP2* loss-of-function animal models (Chao et al., 2010). Our findings show substantial cytoarchitectural disruptions in the MEC pyramidal (L2) and stellate cells (L2) of the RTT mice, emphasizing the function of *Mecp2* in regulating the morphological integrity within the MEC—a region crucial for memory and spatial navigation (Howard et al., 2014). We found that apical dendrites of the pyramidal cells (L2) showed significant shortening in *Mecp*2^+/−^ animals, aligning with earlier research that loss of *Mecp2* impairs dendritic growth and development (Kishi and Macklis, 2004; Chapleau et al., 2009). On the other hand, apical dendritic number remained unaffected, indicating that *Mecp2* functional loss may selectively affect dendritic elongation rather than branching. Even though the overall cellular connectivity may still be impaired, we noticed a localized increase in the apical dendritic intersections close to the soma at 40 μm, suggesting the compensatory dendritic remodeling (Belichenko et al., 2009).

Unlike apical dendrites, the morphology of basal dendrites in pyramidal cells at L2 exhibited increased complexity—as evidenced by the greater dendritic complexity observed between 20 and 60 μm from the soma (domain that integrates cortical inputs) in the *Mecp*2^+/−^ mice. It's well-documented that neurons in the L2 are the major projections toward EC > dentate gyrus > CA2/CA3 areas of the hippocampus, and any morphological changes in L2 neurons may impact EC-hippocampal neural connections (Witter and Amaral, 1991; Witter et al., 2017). In line with this agreement, as the changes mainly happen in the basal region, we suggest that they affect the signals terminating in the L2 rather than those arriving far away in L1, which could impact the overall function within MEC-hippocampus signaling or compensatory remodeling because of RTT pathology (Fukuda et al., 2005). Shrinkage of soma size is associated with neurotrophic or metabolic dysfunctions, particularly in BDNF signaling—a well-recognized downstream target of MeCP2 (Chang et al., 2006). In this study, we observed a reduction in the soma size of pyramidal cells, consistent with the above findings of RTT models.

Another key component of the grid cell network in the MECII region is stellate cells, which regulate the synaptic information to other neurons. In this study, *Mecp*2^+/−^ mice showed modestly reduced mean dendritic length but exhibited dendritic hypertrophy, as evinced by the greater number of primary dendrites in stellate cells at L2. This dendritic reorganization may indicate a malformed developmental trajectory or compensatory sprouting (Belichenko et al., 2009). Increased dendritic intersections from Sholl analysis near 20–50 μm from the soma, again revealing distance-specific remodeling without an increase in total dendritic complexity. Since spines are crucial for synaptic excitatory response, their loss is likely to alter the neuronal connectivity and integrity, leading to several behavioral deficits (Chapleau et al., 2009). In this study, we found that MECII pyramidal and stellate cells exhibited fewer dendritic spines—where synaptic connections form. This reduction in spine density could indicate synaptic dysfunction; such features are commonly reported in the RTT pathology involving MeCP2 dysfunction (Zoghbi, 2003; Chahrour et al., 2008). In contrast to the pyramidal cells, the soma size was significantly enlarged in stellate cells, suggesting cell-type-specific effects of *Mecp2* functional loss. This diverse effect may result from different gene regulation or metabolic demands across several neuronal types and needs future investigations (Chahrour and Zoghbi, 2007).

In summary, pyramidal and stellate cells in the MECII region showed marked structural alterations, including dendritic morphology, soma size and spine density, suggesting that Mecp2 loss-of-function can disrupt synaptic connectivity between the MEC-hippocampal axis (Table 1). Overall, our study highlights how *Mecp2* dysfunction can differentially alter dendritic cytoarchitectures in these MECII cell types, offering insights into the neural basis of spatial and memory dysfunction in RTT pathology.

**Table 1:** *Mecp*2^+/−^ mice exhibits structural changes in their MECII pyramidal and stellate cells.

***Mecp2***	**MECII**						
	**Mean dendritic length**	**Mean dendritic segments**	**Mean intersections**	**Total intersections**	**Mean primary dendrites**	**Mean soma size**	**Mean spine density**
**Pyramidal cells**							
*Apical*	↓ (*p* = 0.001)	# (*p* = 0.719)	↑ @ 40 μm (*p* = 0.029)	# (*p* = 0.532)	NA	↓ (*p* = 0.015)	↓ (*p* = 0.006)
*Basal*	↑ (*p* = 0.032)	↑ (*p* = 0.025)	↑ 20–60 μm [20 μm, *p* = 0.047; 30 μm, *p* = 0.007; 40 μm, *p* = 0.021; 50 μm, *p* = 0.007); 60 μm, *p* = 0.019]	↑ (0.036)	↑ (0.012)		
**Stellate cells**							
	↑ (*p* = 0.044)	# (*p* = 0.760)	↑ from 20–50 μm [20–40 μm, *p* = 0.001); 50 μm, *p* = 0.006]	#(*p* = 0.116)	↑ (*p* = 0.001)	(*p* = 0.001)	↓ (*p* = 0.002)

*p* value shows the statistical significance difference with respective to WT, Mann-Whitney *t*-test (mean ± SEM, *n* = 6). ↑, Increase; ↓, Decrease; #, non-significant; MECII, medial entorhinal cortex II; NA, not applicable; μm, micrometer.

## Data Availability

The original contributions presented in the study are included in the article/Supplementary material, further inquiries can be directed to the corresponding authors.
