# Supplementary material for: Altered dendritic morphology of MEC II pyramidal and stellate cells in Rett syndrome mice
Source: Front Neuroanat. 2025 Jun 24;19:1580435. doi: 10.3389/fnana.2025.1580435 (PMC12236101; doi:10.3389/fnana.2025.1580435)
Supplement: Supplementary file 1 [file Data_Sheet_1.pdf]

Altered dendritic morphology of MEC II pyramidal and stellate cells in Rett syndrome mice

Manigandan Krishnan[1], Ayishal B Mydeen[2], Mohammed M. Nakhal[2], Marwa F. Ibrahim[2], Richard L. Jayaraj[1], Milos R. Ljubisavljevic[3], Mohammad I. K. Hamad[2*], Fatima Y. Ismail[1,4*]

***Correspondence:** fatima.ismail@uaeu.ac.ae and m.hamad@uaeu.ac.ae

This file includes:

Supplementary Text Information

1.0 Supplementary Materials and Methods

2.0 Supplementary Results

3.0 Supplementary Figures

4.0 Supplementary References

1.0 Supplementary Materials and Methods

1.1. *Mecp2* $^{+/-}$ mice breeding, and genotyping

To induce Rett syndrome-like phenotypes in mice, heterozygous female *Mecp2* mice, which possess deletions in exons 3 and 4, were mated with male WT mice to generate the F1 hybrids. Subsequent breeding was conducted within the F1 hybrids to generate all the *Mecp2* genotypes. All littermates were separated according to the sex and genotyped to eliminate potential confounding effects of genetic background unrelated to the *Mecp2* mutation. Female *Mecp2*$^{+/-}$ mice and female WT controls were used in the study.

1.2. Genotyping

The genomic DNA from the tail snip of the newborn pubs was extracted according to the manufacturer's instructions using Wizard® genomic DNA kit (Promega Corporation, Madison, WI, USA). DNA concentrations were measured using a NanoDrop 2000 spectrophotometer (Thermo Fisher Scientific, Marietta, OH, USA). PCR reactions were then performed in a total volume of 20 µL reaction mixture containing 100 ng of template DNA, 1X Go Taq® green master mix (Promega Corporation, Madison, WI, USA) and 1 µM of each primer [Common forward primer (oIMR1436) 5′-GGTAAAGACCCATGTGACCC-3′, mutant reverse primer (oIMR1437) 5′-TCCACCTAGCCTGCCTGTAC-3′, wild type reverse primer (oIMR1438) 5′-GGC TTGCCACATGACAA-3′]. Genotypes were subjected to a touchdown (TD) PCR program, as follows: Thirty seconds of denaturation at 94°C, samples were subjected to a TD program (94°C for 30 s, 63°C for 30 s, and 72°C for 30 s), followed by a 1.0°C drop in an annealing temperature per cycle up to 59°C. Following the TD program, 30 cycles were done (94°C for 30 s, 58°C for 30 s, and 72°C for 30 s), followed by a 10-minute extension at 72°C. Amplification products were run on a 2% agarose gel using Tris-acetate EDTA (TAE) buffer. As shown in Supplementary Figure S1A, heterozygous *Mecp2*$^{+/-}$ female and male littermates exhibited two bands at approximately 400 bp and 416 bp, respectively, while WT mice displayed a single band at 320 bp.

1.3. Behavioral analysis

Neurological anomalies like sensorimotor function were assessed using hind limb clasping, as previously reported (Miedel et al., 2017). *Mecp2*$^{+/-}$ and WT female mice (n = 6, 12 months old) were suspended by their tails for 10 s to induce clasping behavior. The severity of clasping was scored using the following criteria: 0 - hind limbs consistently splayed outward from the abdomen; 1 - one hind limb partially retracted towards the abdomen during observation; 2 - both hind limbs partially retracted towards the abdomen; 3 - hind limbs fully retracted toward the abdomen; and 4 - both hind limbs fully retracted with curled toes and complete immobility.

2.0 Supplementary Results

2.1. Mecp2$^{+/-}$ mice had lower body and brain weight, and characteristic hindlimb clasping

The characteristic features of RTT pathology begin to emerge after postnatal month 10, manifesting as growth retardation and abnormal hindlimb clasping(Guy et al., 2001). To capture these pathological changes, we assessed body weight changes in Mecp2+/- mice at 12 months of age, a time point when RTT symptoms are typically evident. Mecp2+/- mice had significantly lower body weight (18±1.16g, Supplementary Figure S1B) compared to WT controls (27.12±0.71g, $p<0.001$). This reduction in body weight was associated with a significant decrease in brain weight (Supplementary Figure S1C) in Mecp2+/- mice (0.53±0.01g) compared to WT mice (0.63±0.01g). Behavioural analysis revealed that Mecp2+/- mice exhibited severe hindlimb clasping (Supplementary Figure S1D) and had severe hindlimb clasping scores (3.10±0.20, Supplementary Figure S1E) compared to WT mice (0.17±0.17, $p<0.001$), indicating the presence of sensorimotor abnormalities.

3.0. Supplementary Figures

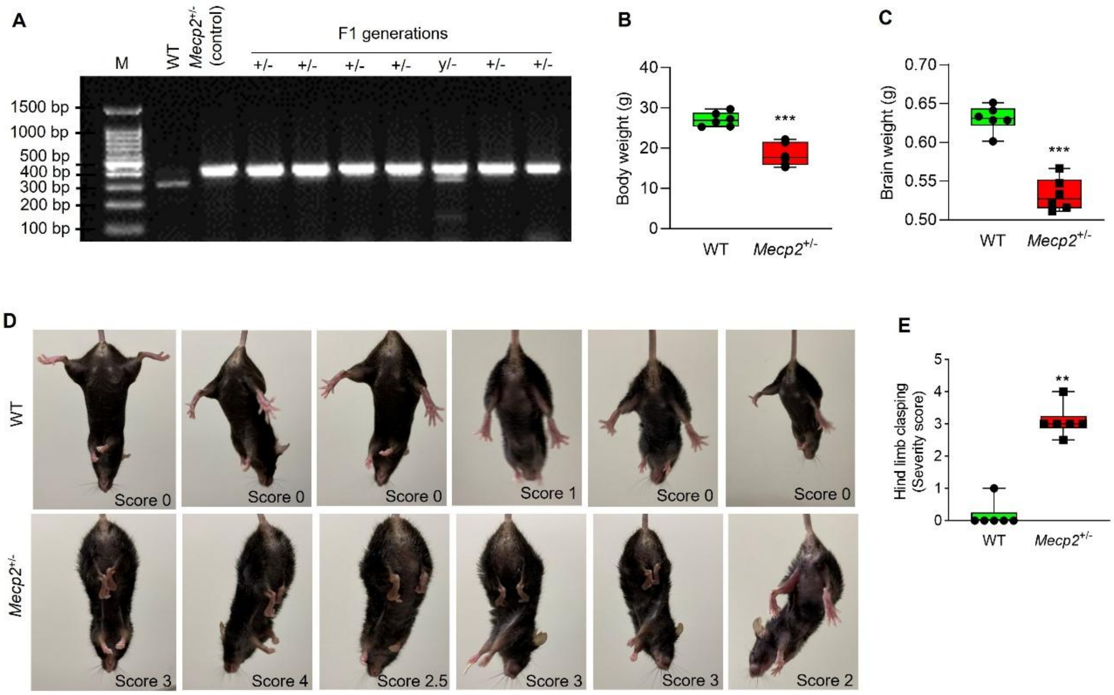

Supplementary Figure S1. *Mecp2*$^{+/-}$ mice exhibit reduced body and brain weight, with characteristic hindlimb clasping. (A) PCR analysis of *Mecp2*$^{+/-}$ female and male littermates. Lane M shows the 100 bp DNA marker, with controls consisting of female wild-type (WT +/+), heterozygous male (-/y) and female (+/-) *Mecp2* mice. Results show the expected genotype with two bands (~400 bp, ~416 bp) for heterozygous mice. (B) body weight, (C) brain weight, (D) hindlimb clasping, and (E) hindlimb clasping score of *Mecp2*$^{+/-}$ mice compared to WT (All values expressed as mean ± SEM; n=6 mice/group; (B, C), $^{***}p<0.001$ by Shapiro-Wilk t-test (B, C); $^{**}p<0.01$ by Mann-Whitney t-test (D)) .

4.0. Supplementary References

Guy, J., Hendrich, B., Holmes, M., Martin, J.E., and Bird, A. (2001). A mouse Mecp2-null mutation causes neurological symptoms that mimic Rett syndrome. *Nat Genet* 27(3), 322-326. doi: 10.1038/85899.

Miedel, C.J., Patton, J.M., Miedel, A.N., Miedel, E.S., and Levenson, J.M. (2017). Assessment of Spontaneous Alternation, Novel Object Recognition and Limb Clasping in Transgenic Mouse Models of Amyloid-beta and Tau Neuropathology. *J Vis Exp* (123). doi: 10.3791/55523.
